# Trends in Karyotype Evolution in *Astyanax* (Teleostei, Characiformes, Characidae): Insights From Molecular Data

**DOI:** 10.3389/fgene.2018.00131

**Published:** 2018-04-16

**Authors:** Rubens Pazza, Jorge A. Dergam, Karine F. Kavalco

**Affiliations:** ^1^Laboratory of Ecological and Evolutionary Genetics, Institute of Biological and Health Sciences, Federal University of Viçosa, Rio Paranaíba, Brazil; ^2^Laboratory of Molecular Systematics “Beagle”, Department of Animal Biology, Federal University of Viçosa, Viçosa, Brazil

**Keywords:** cytotaxonomy, molecular evolution, chromosomal rearrangements, mtDNA, chromosomal symplesiomorphy, chromosomal synapomorphy, chromosomal autapomorphy

## Abstract

The study of patterns and evolutionary processes in neotropical fish is not always an easy task due the wide distribution of major fish groups in large and extensive river basins. Thus, it is not always possible to detect or correlate possible effects of chromosome rearrangements in the evolution of biodiversity. In the *Astyanax* genus, chromosome data obtained since the 1970s have shown evidence of cryptic species, karyotypic plasticity, supernumerary chromosomes, triploidies, and minor chromosomal rearrangements. In the present work, we map and discuss the main chromosomal events compatible with the molecular evolution of the genus *Astyanax* (Characiformes, Characidae) using mitochondrial DNA sequence data, in the search for major chromosome evolutionary trends within this taxon.

## Introduction

The role of chromosomal rearrangements in the evolution of organisms has been a matter of debate for many years. The initial observations that closely related species differ in their karyotypes was later supported by evidence that unbalanced rearrangements can interfere with gametogenesis, decrease gene flow, and reinforce reproductive isolation leading to speciation (Rieseberg, 2001; Navarro and Barton, 2003). On the other hand, some organisms tolerate a certain amount of chromosomal rearrangement, which is often referred to as karyotypic plasticity (Jónsson et al., 2014; Havelka et al., 2016). This complicates the task of explaining the possible role of rearrangements in the evolution of organisms, since the variation may result in speciation or stay as polymorphism within populations.

Since Moreira-Filho and Bertollo (1991), who proposed *Astyanax scabripinnis* as a species complex based on cytogenetic and morphometric data, chromosome variation has been regarded as part of speciation processes in *Astyanax*. Before that, however, it was already acknowledged that some populations could differ in their karyotype formulae and diploid numbers (Morelli et al., 1983). Thus, it is now known that populations currently assigned to *A. scabripinnis* and to the other nominal species are characterized by some degree of inter- or intra-population chromosome variation, with diploid numbers ranging from 46 to 50 chromosomes; such is the case of *Astyanax fasciatus* (Pazza et al., 2006). Molecular cytogenetics (i.e., satellite DNA and ribosomal genes localization) has also provided further taxonomically informative data (for a review, see Pazza and Kavalco, 2007).

Although little is known about the cause and effect of the rearrangements during the evolutionary process, the correlation between independent data sets such as molecular and chromosomal data suggest that some karyotypic signatures are associated with organismic evolution. Based on *Cytochrome B (CytB)* sequences, Mello et al. (2015) found three genetically distinct clades out of 17 *Astyanax* nominal species from the Iguaçu and adjacent river basins. Likewise, using *Cytochrome Oxydase I* (*COI*) sequences, Rossini et al. (2016) found five clades out of 64 nominal species plus 12 provisionally identified taxa. Based on DNA barcoding criteria, these authors identified only 21 morphological species. These studies also suggest the possibility of horizontal transfer that affects some species within otherwise cogent taxa. Indeed, hybridization may play a fundamental role in the genus speciation, especially when these events involve chromosomal characteristics. The matter is particularly difficult since the claims of possible natural hybrids in the specialized literature are rare (Artoni et al., 2006; Pazza et al., 2006; Sassi et al., 2018).

Thus, we analyzed mitochondrial sequences from individuals with known chromosome characteristics within the five clades proposed by Rossini et al. (2016) to describe the relations between DNA sequences and chromosomal rearrangements. We obtained a phylogenetic tree that depicts chromosomal characteristics that are strongly associated with the proposed clades.

## Materials and Methods

In the present study we sequenced samples of 195 individuals from 16 nominal species of the genus *Astyanax*, deposited in the tissue collection of the Laboratory of Ecological and Evolutionary Genetics (LaGEEvo), of the Federal University of Viçosa, Rio Paranaíba campus. The species were chosen based on their chromosomal data as analyzed in previous works and because they were included in Rossini et al. (2016) clades. Geographic coordinates, Vouchers, and GenBank sequence access numbers are summarized in Supplementary Table S1. This study was carried out in accordance with the recommendations of the Guide for the Care and Use of Laboratory Animals by the Conselho Nacional de Controle de Experimentação Animal (CONCEA). Tissue samples of fish of the genus *Astyanax* deposited in the tissue bank of the Laboratory of Ecological and Evolutionary Genetics (LaGEEvo). All the specimens used had their chromosomal data analyzed in previous works. No additional animals were sacrificed to this study.

Our hypothesis on chromosome evolution was based on the following chromosome characters: diploid numbers, fundamental number (FN), location of 5S DNA ribosomal sites, presence/absence of a 5S rDNA site in a specific submetacentric, referred to as the “marker” chromosome by Almeida-Toledo et al. (2002) and Kavalco et al. (2005), and finally, the amount and distribution of the repetitive DNA As-51 probe (Mestriner et al., 2000).

Total DNA extraction from muscle or liver samples was carried out using commercial kits (PureLink Genomic DNA minikit, Invitrogen^TM^), according to the manufacturer’s instructions. Amplification of mitochondrial (mtDNA) subunits 6 and 8 of the *ATP synthase* enzyme gene (*ATPase 6/8*) was accomplished using the primers ATP8.2-L8331 (5′-AAAGCRTTRGCCTTTTAAAGC-3′) and CO3.2-H9236 (5′-GTTAGTGGTCAGGGCTTGGRTC-3′) (Sivasundar et al., 2001). PCR was performed in a final volume of 25 μL, with 2.5 μL of 10× Taq buffer, 1 μL of MgCl_2_, 1 μL of each primer, 0.2 μL Taq DNA polymerase, 12.8 μL ultrapure water, 1.5 μL of dNTP, and 5 μL of DNA. The amplification reactions were performed in a thermocycler at 95°C for initial denaturation (2 min) and 30 cycles of 94°C (30 s), 58°C (30 s), and 72°C (1 min). The PCR product was visualized on 1% agarose gel; purification and sequencing was performed by a third-party company (Macrogen, Korea).

Sequence editing was performed using Chromas Lite v2.01 and sequence identity was checked with BLASTn^1^. Sequences alignment was carried out with ClustalW v1.6 (Thompson et al., 1994) as implemented in MEGA v7 (Kumar et al., 2016). A Maximum Likelihood was obtained using the best model fit with MEGA v7 (Kumar et al., 2016), and chromosome characters were plotted on this ML phylogram. Phylogenetic signal was estimated using bootstrap (Felsenstein, 1985).

## Results

**FIGURE 1:**
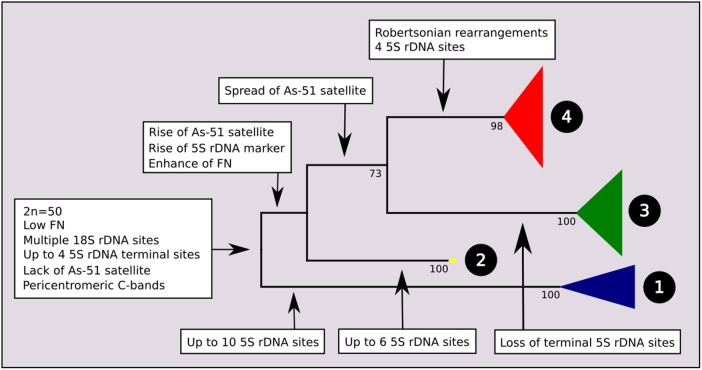
Molecular Phylogenetic analysis by Maximum Likelihood Method (MCL) with synapomorphies of each clade. The evolutionary history was inferred by using the Maximum Likelihood method based on the Tamura 3-parameter model. The tree with the highest log likelihood (–3974.2120) is shown. Initial tree(s) for the heuristic search were obtained automatically by applying Neighbor-Join and BioNJ algorithms to a matrix of pairwise distances estimated using the MCL approach, and then selecting the topology with superior log likelihood value. A discrete Gamma distribution was used to model evolutionary rate differences among sites [five categories (+*G*, parameter = 0.4001)]. The condensed tree is drawn to scale, with branch lengths measured in the number of substitutions per site. The analysis involved 196 nucleotide sequences. All positions containing gaps and missing data were eliminated. There were a total of 531 positions in the final dataset. Evolutionary analyses were conducted in MEGA7. Value of bootstrap related to 500 replicates is below of branches.

A total of 195 mitochondrial DNA sequences were obtained from individuals with known chromosomal characteristics, corresponding to 16 nominal species of *Astyanax* from the Neotropical region. The *ATPase 8* gene yielded a 530 bp partial sequence without insertions, deletions, or stop codons, and the substitution model was Tn93 + G. The maximum likelihood phylogram indicated four main clades with strong bootstrap support. The main events of chromosome differentiation were plotted on a simplified phylogenetic tree (**Figure 1**) according to the trends observed in the analyzed specimens and data from the literature. At its root, and according to the chromosomal characteristics of closely related species, we propose a most recent common ancestor with a karyotype with 2n = 50 chromosomes, low FN, one or two pairs of chromosomes bearing 5S ribosomal rDNA sites at the terminal region, and multiple 18S rDNA sites.

### Clade 1

**FIGURE 2:**
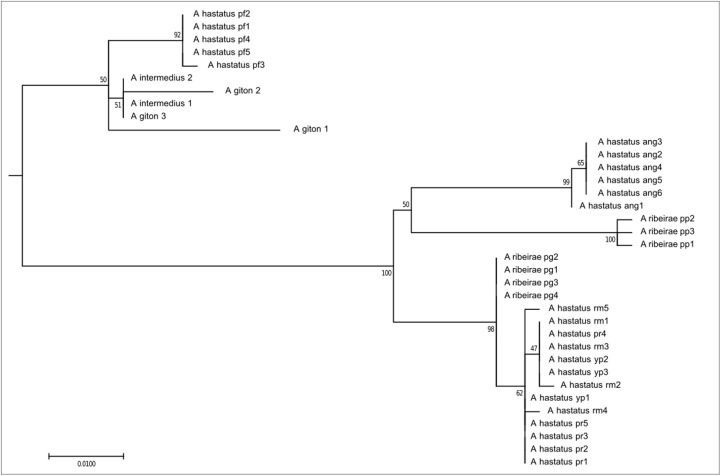
Molecular Phylogenetic of the Clade 1 analysis by Maximum Likelihood Method. Parameters described in **Figure 1**. Clade 1 is formed by species distributed only in coastal drainages as *A. hastatus*, *A. ribeirae*, *A. intermedius*, and *A. giton*.

The first clade, composed of 36 individuals, corresponds to exclusively coastal species, here represented here by *A. ribeirae*, *A. intermedius*, *A. giton*, and *A. hastatus* (**Figure 2**), which have been recently proposed as members of the Probolodini (Silva, 2017). In this group, all individuals have 2n = 50 chromosomes; low FN; absence of repetitive DNA As51 and the 5S rDNA marker chromosome; little constitutive heterochromatin, distributed mainly in the pericentromeric region; a greater number of 5S rDNA sites (ranging from 6 to 10 sites) distributed in terminal regions; and a variable number of Ag-NORs and 18S rDNA sites.

### Clade 2

**FIGURE 3:**
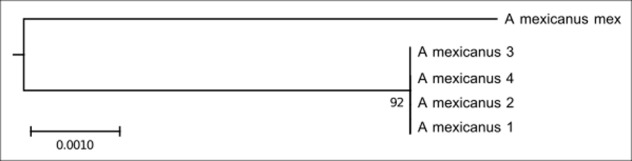
Molecular Phylogenetic of the Clade 2 analysis by Maximum Likelihood Method. Parameters described in **Figure 1**. Clade 2 is formed by *A. mexicanus*.

This clade is composed of five individuals of *Astyanax mexicanus* (**Figure 3**). This species has a few repetitive As51 DNA sites and 5S rDNA is distributed on six sites, including the submetacentric, marker chromosome pair.

### Clade 3

**FIGURE 4:**
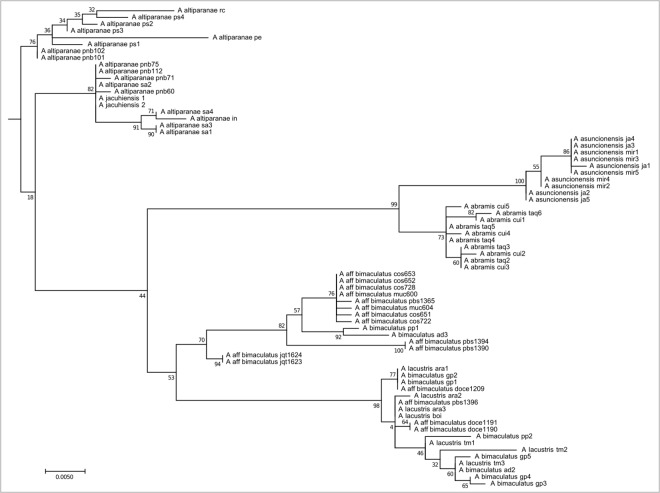
Molecular Phylogenetic of the Clade 3 analysis by Maximum Likelihood Method. Parameters described in **Figure 1**. Clade 3 is formed by species with humeral oval spot like the *A. bimaculatus* complex and the related species.

This clade comprises 71 individuals with oval humeral spots distributed in coastal, Upper Paraná, Paraguay, and São Francisco river basins, belonging to the *Astyanax bimaculatus* species complex, such as *A. altiparanae*, *A.* aff. *bimaculatus*, *A. lacustris*, *A. assuncionensis*, and *A. abramis* (**Figure 4**). They all have 50 chromosomes, with a predominance of submetacentric chromosomes, and consequently high FN numbers. The presence of As51 repetitive DNA in a few sites and the presence of 5S rDNA restricted to the submetacentric marker chromosome is remarkable. The distribution of heterochromatin and rDNA 18S/Ag-NORs is variable.

### Clade 4

**FIGURE 5:**
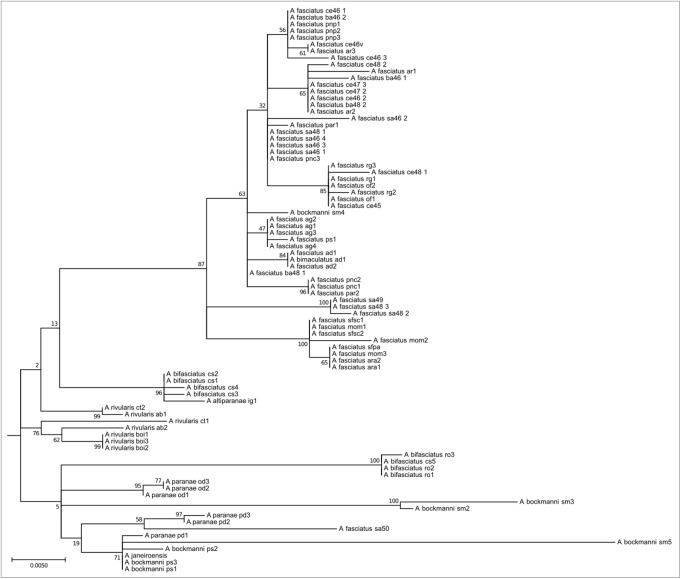
Molecular Phylogenetic of the Clade 4 analysis by Maximum Likelihood Method. Parameters described in **Figure 1**. Clade 4 is formed by red-tailed species and their related group as *A. fasciatus*, the *A. scabripinnis* group, *A. bockmanni*, and *A. bifasciatus*. This group is the most chromosome diverse in the genus and shows little mitochondrial divergence.

This clade encompasses species widely distributed in the Upper Paraná, São Francisco, and coastal river basins. Eighty four individuals of *A. paranae*, *A. rivularis*, *A. bifasciatus*, *A. fasciatus*, and *A. bockmanni* are represented in this clade (**Figure 5**). Species of this clade present high intra and interspecific chromosome variability, with 2n = 46 to 2n = 50 chromosomes. Chromosomes carrying As51 satellite DNA sites vary from none to 14 sites. Most species have four 5S rDNA sites, which may include the chromosomal marker pair. As in Clade 3, they have FN numbers, relatively few acrocentric chromosomes, 18S rDNA sites and variable Ag-NORs, and highly variable constitutive heterochromatin distribution.

## Discussion

The phylogram obtained in the present work using the mitochondrial DNA sequence (*ATPase* subunit 6) is mostly congruent with that obtained by Mello et al. (2015) using the mitochondrial DNA sequence of the *cytochrome b* gene, as well as that obtained by Rossini et al. (2016) using the mitochondrial *COI* sequence. Karyotypical data allow to give further support to at least some monophyletic groupings within the current genus *Astyanax*, corresponding to the major clades, as it was proposed by Travenzoli et al. (2015) in Bryconidae. Despite this cohesion, some species seem to have haplotypes distributed in different clades, as demonstrated by Rossini et al. (2016). According to the authors, the *COI* sequence is not an appropriate tool to recover phylogenies, but rather to identify species (Hebert and Gregory, 2005). For the *Astyanax* genus, this sequence also seems to be unsuitable for species identification, since of the more than 70 nominal species analyzed by Rossini et al. (2016), only 21 were unequivocally identified by barcoding. The low genetic distances observed in the mitochondrial analyses among species of the *Astyanax* genus are often explained by the rapid divergence between them (Ornelas-García et al., 2008; Carvalho et al., 2011; Rossini et al., 2016). Therefore, multidisciplinary approaches may be more effective for reconstructing phylogenies within this genus. In lower systematic levels, molecular data associated with the chromosomal features were particularly effective to understand evolutionary patterns in *A.* aff. *bimaculatus* (Kavalco et al., 2011) and *A. fasciatus* (Pansonato-Alves et al., 2013; Kavalco et al., 2016).

Overlapping cytogenetic onto molecular phylograms offers an insight on large-scale chromosome evolutionary *Astyanax*. This analysis allows us to point out as the main specific chromosomal markers to be explored: the distribution patterns of 5S rDNA and As51 repetitive DNA, as they show signatures within the genus *Astyanax*. On the other hand, other characters such as location of the Nuclear Organizing Regions and C-banding shows patterns seem more informative at population level, as demonstrated by Jacobina et al. (2011) in *Hoplias malabaricus* (Erythrinidae). Although other markers have been used sporadically in chromosome studies of the genus *Astyanax* (Barbosa et al., 2015, 2017; Silva et al., 2015; Piscor and Parisi-Maltempi, 2016), their informative value will require intensive sampling in an array of species.

The genus *Astyanax* is currently *incertae sedis* in the Characidae family, and the evolutionary relations within the genus, as well as its phylogenetic relation to other genera of the family are quite challenging both from the morphological point of view (Mirande, 2009, 2010), and from the molecular systematic (Oliveira et al., 2011; Rossini et al., 2016). Despite the incomplete database, others genera related to *Astyanax* usually show more conservative cytogenetic characters, such as steady patterns of 50 chromosomes, multiple NORs and 5S ribosomal genes located in one or more pairs of chromosomes at the terminal region. These suite of characteristics have been reported in the genus *Oligosarcus* (Kavalco et al., 2005; Hattori et al., 2007; Barros et al., 2015), *Deuterodon* (Mendes et al., 2011; Coutinho-Sanches and Dergam, 2015), *Hollandichthys* and *Ctenobrycon* (Carvalho et al., 2002), plus the absence of the homology with the As51 repetitive DNA (Kavalco et al., 2005), suggesting that these characters are close to the evolutionary origins of *Astyanax*, being symplesiomorphies in clade 1.

### Clade 1

The clade 1 obtained by our molecular analyses corresponds to clade 5 of Rossini et al. (2016). In the specimens analyzed we can observe the plesiomorphic chromosome characteristics (as a karyotype with 2n = 50 chromosomes and a low FN) and with good structure in *A. ribeirae* (Kavalco et al., 2010), *A. intermedius*, and *A. giton* (Kavalco and Moreira-Filho, 2003; Kavalco et al., 2004). In *A. hastatus*, different cytotypes with 2n = 50 chromosomes have been observed, constituting yet another species complex within the genus *Astyanax* (Kavalco et al., 2009), as corroborated by the present study (**Figure 2**). Also, a combination of molecular and chromosomal data suggests that the current *A. hastatus* may encompass more than one OTU (operational taxonomic unit) and so, more than one ESU (evolutionarily significant unit). The *Astyanax* species distributed in the coastal basins were added to the Probolodini group with species from the genus *Probolodus*, *Deuterodon*, and *Myxiops* and *Hyphessobrycon luetkenii* (Silva, 2017). These other species are less studied by cytogenetic methods, but until now, the chromosome characteristics seems to be shared (Mendes et al., 2011; Coutinho-Sanches and Dergam, 2015), bringing new evidence supporting the group. Unfortunately, As51 repetitive DNA in these coastal distribution species has not been confirmed so far (Kavalco et al., 2007, 2009).

Smaller variations in the number of 5S rDNA sites can be observed among these species, although they are always located in the proximal or distal region of acrocentric chromosomes, reaching up to 10 markings in *A. intermedius* and *A. giton*, which differ by a pericentric inversion (Kavalco et al., 2004). The hypothesis that these characteristics are a symplesiomorphy can be corroborated in independent chromosome data, such as those obtained for *A. taeniatus* (Cunha et al., 2016), which is also included in the same clade 5 by Rossini et al. (2016). The evolutionary dynamics of this gene are related not only to variations in non-transcribed spacers, but also to syntenia with long and short interspersed nuclear elements, non-long terminal repeat retrotransposons, U-snRNA families, and microsatellite polymorphisms (Rebordinos et al., 2013). According to these authors, polymorphisms in non-transcribed regions are observed in fish. Polymorphisms in transcribed regions do not appear to interfere with the cellular activity of 5S rDNA, and the molecular diversity of the 5S rDNA gene families is greater than the chromosome diversity (Rebordinos et al., 2013).

### Clade 2

At the root of clades 2, 3, and 4, it is possible to hypothesize two main chromosomal events: the occurrence one of the 5S rDNA sites to the proximal position of a specific submetacentric chromosome pair, which has been considered as a marker (Almeida-Toledo et al., 2002) and the presence of As51 repetitive DNA in the *Astyanax* genome.

The 5S rDNA in clade 2 appears on a pair of two-arm chromosomes in the proximal region of the centromere (Kavalco and Almeida-Toledo, 2007). This marker may have arisen by pericentric inversion from an acrocentric chromosome bearing the 5S rDNA site, which should represent the most basal character state.

In addition to this site, *A. mexicanus* also presents four more 5S rDNA sites on acrocentric chromosomes, being distal markings on one pair and proximal markings on another (Kavalco and Almeida-Toledo, 2007). The isolation of the group represented by the *A. mexicanus* is congruent with some molecular data that relate ichthyofauna invasions in Central America with the genesis of the Panamá Isthmus (around 3.3 Mya; Ornelas-García et al., 2008), as well as with clustering with DNA barcoding (Rossini et al., 2016). Unfortunately, cytogenetic data for the species of this group are scarce, but it would not be surprising to have independent autapomorphies of the karyotype evolution of the cisandine species of *Astyanax*.

As51 satellite DNA is partly repetitive tandem DNA and holds similarities with transposable elements that were isolated from a population of *A. scabripinnis* carrier of chromosome B (Mestriner et al., 2000). In this population, this DNA was located mainly in the B chromosome, besides two to four sites in the distal region of acrocentric chromosomes. Considering the possible origin from transposable elements, it is appropriate to assume that their distribution was initially restricted to a few sites and subsequently spread in the genome by intrinsic mechanisms of the repetitive sequence amplification. In fact, the individuals of *A. mexicanus* that form clade 2 in the present work have few sites that carry this satellite DNA, and that it is shown more diffusely than in other species, which suggests a smaller number of copies or even only partial homology (Kavalco and Almeida-Toledo, 2007). This divergence might be related to independent evolution of *A. mexicanus* relative to the *A. scabripinnis* strain that donated this probe.

### Clade 3

The increase of As51 satellite DNA sites characterizes the common ancestral root of clades 3 and 4, in contrast to the loss of 5S rDNA sites on acrocentric chromosomes observed in members of Clade 3. Clade 3 is composed of the species complex “*A. bimaculatus*,” represented herein by *A. altiparanae*, *A.* aff. *bimaculatus*, *A. lacustris*, *A. asuncionensis*, and *A. abramis*. Specimens from this clade present 2n = 50 chromosomes, with a higher FN, multiple Nucleolar Organizing Regions, and consequently multiple sites of 18S rDNA, according to the samples analyzed (Kavalco et al., 2011) and others independent studies (Almeida-Toledo et al., 2002; Fernandes and Martins-Santos, 2004, 2006; Peres et al., 2008; Hashimoto et al., 2011; Giongo et al., 2013).

Clade 3 is characterized by a synapomorphy: only one pair of chromosomes carrying a rDNA 5S site on its pericentromeric region (Kavalco et al., 2011) and in the literature (Almeida-Toledo et al., 2002; Fernandes and Martins-Santos, 2006; Peres et al., 2008, 2012; Hashimoto et al., 2011; Paiz et al., 2015). One exception is *A. abramis*, which presents in addition to the site of the chromosomal marker pair, a pair of extra-acrocentric chromosomes with proximal marking (Paiz et al., 2015; Piscor et al., 2015). This differentiated pattern may be an autapomorphy of *A. abramis*, whose molecular distinctiveness within the group was also evident in our analysis (**Figure 5**). Additionally, the pattern of occurrence of a 5S rDNA carrier pair can be seen in other species related to the *A. bimaculatus* group, as in *A. janeiroensis* (Vicari et al., 2008), *A. goyacensis* (Santos et al., 2013), and *A. elachylepis* (Santos et al., 2016).

In turn, there is little data on the distribution of As51 satellite DNA in this group, besides previous reports (Kavalco et al., 2011). In these species, small sites are observed, suggesting relatively lower number of *in tandem* copies and more restricted distribution through the karyotype, except for *A. bimaculatus* from coastal basins, in which As51 satDNA is absent (Kavalco et al., 2011). On the other hand, in *Astyanax janeiroensis*, a considerable number of very conspicuous sites are observed (Vicari et al., 2008; Kantek et al., 2009b). Although the Catalog of Fishes (Eschmeyer et al., 2017) characterizes the distribution of *A. janeiroensis* as being “in Brazil”, Melo (2001) suggests that its distribution is restricted to the basins of the Paraíba do Sul and other coastal drainages. These discrepant and independent occurrences in *A. janeiroensis* and *A. bimaculatus* can be a result of the historical biogeography of coastal drainages, affected by stream capture from continental basins and complex dispersal due to marine regressions and transgressions (Pereira et al., 2013). Fish faunas in these regions are characterized by a high degree of endemism and low species richness (Albert and Reis, 2011).

### Clade 4

Clade 4 comprises a group of species with the highest chromosomal variability in the genus, with 2n ranging from 46 to 50 chromosomes and including the species complexes *A. scabripinnis* (encompassing *A. rivularis* and *A. paranae*) and *A. fasciatus*, as well the species *A. bockmanni* and *A. bifasciatus* (formerly referred to as *Astyanax* sp. B). Equally variable among the species of this clade is the number of NOR/rDNA 18S sites and the constitutive heterochromatin distribution patterns. The molecular divergence among these species is apparently quite recent, with low genetic distance indices as observed in the clade 1 and by Rossini et al. (2016) using the *COI* gene. Despite some level of structuring, low bootstrap indexes preclude further hypotheses on the genetic diversification of the group. There is no detailed chromosome information on the other species analyzed by Rossini et al. (2016) belonging to this clade, except for *A. parahybae* (Kavalco and Moreira-Filho, 2003; Kavalco et al., 2004) presenting 2n = 48 chromosomes, and *A. schubarti* (Morelli et al., 1983) presenting 2n = 36 chromosomes. Unfortunately, we were unable to obtain the mitochondrial DNA sequence from *A. parahybae* in the present work.

Among the specimens analyzed, this clade presents some species with a conserved chromosome number, always with 2n = 50 chromosomes, such as *A. bockmanni* and *A. bifasciatus* (Fazoli et al., 2003; Kavalco et al., 2009; Hashimoto and Porto-Foresti, 2010). On the other hand, the others species present high levels of numerical chromosome variation.

This diploid number (2n = 50) was also observed in the *A. paranae* specimens from the Paranaíba and some *A. rivularis* specimens from the São Francisco river basin, although other specimens of the Paranaíba river basin also had 2n = 46 chromosomes (data not shown, in preparation). Both species belong to the historical *A. scabripinnis* species complex, characterized by broad sympatric and allopatric karyotype variation (Moreira-Filho and Bertollo, 1991, among others).

Finally, a diploid number that varies from 2n = 46 or 2n = 48 was observed among the analyzed specimens of *A. fasciatus* (Pazza et al., 2006; Kavalco et al., 2016) that was also observed in other populations (Artoni et al., 2006; Medrado et al., 2008; Pansonato-Alves et al., 2013). This variation is considered common in the species, although 2n = 50 chromosomes have already been reported (Artoni et al., 2006).

In relation to rDNA 5S, most of the species/populations already analyzed have the same following pattern: one site on the marker chromosome plus a pair of acrocentric chromosomes with a rDNA 5S site in the proximal region; this was also observed in the specimens analyzed in the present work (Kavalco et al., 2004, 2007, 2009, 2016; Pazza et al., 2006) and in the literature available for *A. bockmanni*, *A. fasciatus*, and *A. parahybae* (Almeida-Toledo et al., 2002; Hashimoto et al., 2011; Silva et al., 2013; Daniel et al., 2015). Despite this relatively conserved pattern, there are two autapomorphies in the 5S rDNA phenotype in *A. fasciatus*. Medrado et al. (2015) reported the occurrence of a small variation in the distribution of 5S rDNA sites from a population of *Astyanax* aff. *fasciatus* where the marker site of the metacentric chromosome is absent, an evident populational autapomorphy. We also considered the additional occurrence of two 5S rDNA sites of *A.* aff. *fasciatus* from the Paraíba do Sul river basin an autapomorphy. In total, six 5S rDNA-bearing chromosomes are detected, represented always by the marker chromosome pair plus two st/a chromosomes pairs in this sample (Kavalco et al., 2016).

In relation to the *A. scabripinnis* group, the most studied from the cytogenetic point of view (Pazza and Kavalco, 2007), several populations of the Coastal, São Francisco, and Upper Paraná rivers show the same mentioned pattern (Mantovani et al., 2005; Fernandes and Martins-Santos, 2006; Peres et al., 2008; Vicari et al., 2008). However, other studies have already demonstrated, in addition to these standard sites, more distal or proximal sites in other pairs of acrocentric chromosomes in populations of coastal rivers and the Upper Paraná basin (Kavalco et al., 2004).

Although absent in our analyses, *A. schubarti* is a species that has available chromosome data and is found in clade 1 of Rossini et al. (2016), the clade analogous to clade 4 of this work. Its low chromosomal number (2n = 36) and high FN suggest the occurrence of Robertsonian events of chromosome fusion at its origin (Morelli et al., 1983). In fact, this species shows the 5S rDNA sites located in the proximal region of two pairs of metacentric chromosomes (Almeida-Toledo et al., 2002). Interestingly, *A. currentinus*, a species recently described (Mirande et al., 2015), presents 2n = 36 chromosomes and the same distribution pattern of *A. schubarti* 5S rDNA, with an additional odd site (Paiz et al., 2015). The authors suggest that these species may belong to the same morphological group and are phylogenetically related. Unfortunately, there are no available phylogenetic data to support this hypothesis.

Among the specimens used in the present study, populations of the *A. fasciatus* and *A. scabripinnis* species complexes presented sites with homology with the As51 satellite DNA (Kavalco et al., 2007, 2013; Pazza et al., 2008). On the other hand, this probe showed no homology in the chromosomes of *A. bockmanni* (Kavalco et al., 2009). Among the species included in this group and in the clade 1 of Rossini et al. (2016), in only one population of *A. rivularis* (cited as *A. scabripinnis*) this repetitive sequence is absent (Abel et al., 2006). On the other hand, other species belonging to clade 1 of Rossini et al. (2016) have already presented homology with As51 satellite DNA, such as *A. parahybae* (Kavalco et al., 2007) and *A. serratus* (quoted as *Astyanax sp.*; Kantek et al., 2009a). Among these species, the available data are mainly concentrated in the group *A. scabripinnis*/*paranae*/*rivularis* (Abel et al., 2006; Kantek et al., 2009b; Barbosa et al., 2015, 2017), and in *A. fasciatus* (Abel et al., 2006; Kantek et al., 2009b; Medrado et al., 2015). The distribution of this satellite DNA in *A. fasciatus* seems to follow a biogeographic pattern, with an increase in the number of sites in drainage populations in the interior of the continent, and a decrease in coastal populations (Kavalco et al., 2013; Medrado et al., 2015). This pattern does not appear to be the same in the *A. scabripinnis* group and its cryptic species.

The absence of a clear biogeographic pattern of satellite DNA distribution in the *A. scabripinnis* populations should be related to the fact that these fish inhabit isolated headwaters. In addition to facilitating the occurrence of vicariance processes, this ecology keeps low levels of gene flow among, with consequent reduction of population sizes and possible strong effect of genetic drift, resulting in random of As51 satDNA. This could easily generate the biogeographic gaps that occur in the genomic distribution and the existence of populations in which this repetitive DNA is absent, even being a basal characteristic for the group. In fact, it is not yet proven if there is any adaptive role for this DNA, even though we have evidence that it is present in a larger amount in the species with the greatest chromosome diversity, with extensive polymorphisms such as *A. fasciatus*. The occurrence of the As51 satDNA predominantly in supernumerary chromosomes (Mestriner et al., 2000) may imply exogenous origin.

The data of the literature obtained in the present study still cannot answer definitively the questions about the origin and evolution of these sequences within the lineages of *Astyanax*, only to indicate trends within specific clades. Can As51 satDNA promote the chromosomal rearrangements that generate the diversity in diploid numbers and karyotype formulas observed in *Astyanax*; or can the genome of species with chromosomal plasticity, such as those of the genus, facilitate the dispersion of repetitive DNAs? To answer this question, we need more information about the role of this DNA in the karyotype and organismic evolution of the group. Fortunately, the genus *Astyanax*, due to the great concentration of studies, is an excellent model to answer these questions of comparative genomics.

## Author Contributions

RP and KK designed the research and performed the analyses. All the authors collected data and contributed to the writing and review of the manuscript.

## Conflict of Interest Statement

The authors declare that the research was conducted in the absence of any commercial or financial relationships that could be construed as a potential conflict of interest.

## Footnotes

1 http://www.ncbi.nlm.nih.gov
