# Supplementary material for: Trends in Karyotype Evolution in Astyanax (Teleostei, Characiformes, Characidae): Insights From Molecular Data
Source: Front Genet. 2018 Apr 16;9:131. doi: 10.3389/fgene.2018.00131 (PMC5911472; doi:10.3389/fgene.2018.00131)
Supplement: Supplementary file 1 [file Table_1.docx]

Table 1 – Hydrographic basin, GPS data and GenBank access of sample used in the phylogenetic analysis.

| Sample | Hydrographic basin | Latitude | Longitude | GenBank |
| --- | --- | --- | --- | --- |
| A abramis cui1 | PRB | -15.70227 | -56.11118 | MH158811 |
| A abramis cui2 | PRB | -15.70227 | -56.11118 | MH158812 |
| A abramis cui3 | PRB | -15.70227 | -56.11118 | MH158813 |
| A abramis cui4 | PRB | -15.70227 | -56.11118 | MH158814 |
| A abramis cui5 | PRB | -15.70227 | -56.11118 | MH158815 |
| A abramis taq2 | PRB | -18.52764 | -54.73934 | MH158816 |
| A abramis taq3 | PRB | -18.52764 | -54.73934 | MH158817 |
| A abramis taq4 | PRB | -18.52764 | -54.73934 | MH158818 |
| A abramis taq5 | PRB | -18.52764 | -54.73934 | MH158819 |
| A abramis taq6 | PRB | -18.52764 | -54.73934 | MH158820 |
| A altiparanae in | PRB | -23.0942 | -47.2606 | MH158821 |
| A altiparanae pe | PRB | -21.30012 | -50.13678 | MH158822 |
| A altiparanae pnb101 | PRB | unknown | unknown | MH158823 |
| A altiparanae pnb102 | PRB | unknown | unknown | MH158824 |
| A altiparanae pnb112 | PRB | unknown | unknown | MH158825 |
| A altiparanae pnb60 | PRB | unknown | unknown | MH158826 |
| A altiparanae pnb71 | PRB | unknown | unknown | MH158827 |
| A altiparanae pnb75 | PRB | unknown | unknown | MH158828 |
| A altiparanae ps1 | PRB | -23.81258 | -47.70878 | MH158829 |
| A altiparanae ps2 | PRB | -23.81258 | -47.70878 | MH158830 |
| A altiparanae ps3 | PRB | -23.81258 | -47.70878 | MH158831 |
| A altiparanae ps4 | PRB | -23.81258 | -47.70878 | MH158832 |
| A altiparanae rc | PRB | -23.00096 | -48.00006 | MH158833 |
| A altiparanae sa1 | PRB | -23.52329 | -45.86255 | MH158834 |
| A altiparanae sa2 | PRB | -23.52329 | -45.86255 | MH158835 |
| A altiparanae sa3 | PRB | -23.52329 | -45.86255 | MH158836 |
| A altiparanae sa4 | PRB | -23.52329 | -45.86255 | MH158837 |
| A asuncionensis ja1 | PRB | -15.70227 | -56.11118 | MH158838 |
| A asuncionensis ja2 | PRB | -15.70227 | -56.11118 | MH158839 |
| A asuncionensis ja3 | PRB | -15.70227 | -56.11118 | MH158840 |
| A asuncionensis ja4 | PRB | -15.70227 | -56.11118 | MH158841 |
| A asuncionensis ja5 | PRB | -15.70227 | -56.11118 | MH158842 |
| A asuncionensis mir1 | PRB | -16.05853 | -57.70761 | MH158843 |
| A asuncionensis mir2 | PRB | -16.05853 | -57.70761 | MH158844 |
| A asuncionensis mir3 | PRB | -16.05853 | -57.70761 | MH158845 |
| A asuncionensis mir4 | PRB | -16.05853 | -57.70761 | MH158846 |
| A asuncionensis mir5 | PRB | -16.05853 | -57.70761 | MH158847 |
| A bifasciatus cs1 | PRB | -25.13484 | -53.36057 | MH158848 |
| A bifasciatus cs2 | PRB | -25.13484 | -53.36057 | MH158849 |
| A bifasciatus cs3 | PRB | -25.13484 | -53.36057 | MH158850 |
| A bifasciatus cs4 | PRB | -25.13484 | -53.36057 | MH158851 |
| A bifasciatus cs5 | PRB | -25.13484 | -53.36057 | MH158852 |
| A bifasciatus ro1 | PRB | -25.15527 | -53.32865 | MH158853 |
| A bifasciatus ro2 | PRB | -25.15527 | -53.32865 | MH158854 |
| A bifasciatus ro3 | PRB | -25.15527 | -53.32865 | MH158855 |
| A bimaculatus ad1 | SEC | -24.3113 | -47.89213 | MH158856 |
| A bimaculatus ad2 | SEC | -24.3113 | -47.89213 | MH158857 |
| A bimaculatus ad3 | SEC | -24.3113 | -47.89213 | MH158858 |
| A bimaculatus gp1 | SEC | -22.46395 | -42.65473 | MH158859 |
| A bimaculatus gp2 | SEC | -22.46395 | -42.65473 | MH158860 |
| A bimaculatus gp3 | SEC | -22.46395 | -42.65473 | MH158861 |
| A bimaculatus gp4 | SEC | -22.46395 | -42.65473 | MH158862 |
| A bimaculatus gp5 | SEC | -22.46395 | -42.65473 | MH158863 |
| A bimaculatus pp1 | SEC | -24.59341 | -47.8776 | MH158864 |
| A bimaculatus pp2 | SEC | -24.59341 | -47.8776 | MH158865 |
| A bockmanni ps1 | PRB | -23.81258 | -47.70878 | MH158866 |
| A bockmanni ps2 | PRB | -23.81258 | -47.70878 | MH158867 |
| A bockmanni ps3 | PRB | -23.81258 | -47.70878 | MH158868 |
| A bockmanni sm2 | PRB | -23.91238 | -47.96125 | MH158869 |
| A bockmanni sm3 | PRB | -23.91238 | -47.96125 | MH158870 |
| A bockmanni sm4 | PRB | -23.91238 | -47.96125 | MH158871 |
| A bockmanni sm5 | PRB | -23.91238 | -47.96125 | MH158872 |
| A fasciatus ad1 | SEC | -24.3113 | -47.89213 | MH158873 |
| A fasciatus ad2 | SEC | -24.3113 | -47.89213 | MH158874 |
| A fasciatus ag1 | PRB | -23.54973 | -48.51238 | MH158875 |
| A fasciatus ag2 | PRB | -23.54973 | -48.51238 | MH158876 |
| A fasciatus ag3 | PRB | -23.54973 | -48.51238 | MH158877 |
| A fasciatus ag4 | PRB | -23.54973 | -48.51238 | MH158878 |
| A fasciatus ar1 | PRB | -22.38323 | -47.43041 | MH158879 |
| A fasciatus ar2 | PRB | -22.38323 | -47.43041 | MH158880 |
| A fasciatus ar3 | PRB | -22.38323 | -47.43041 | MH158881 |
| A fasciatus ara1 | SFR | -20.12317 | -45.98089 | MH158882 |
| A fasciatus ara2 | SFR | -20.12317 | -45.98089 | MH158883 |
| A fasciatus ba46 1 | PRB | -21.92695 | -47.3673 | MH158884 |
| A fasciatus ba46 2 | PRB | -21.92695 | -47.3673 | MH158885 |
| A fasciatus ba48 | PRB | -21.92695 | -47.3673 | MH158886 |
| A fasciatus ba48 2 | PRB | -21.92695 | -47.3673 | MH158887 |
| A fasciatus ce45 | PRB | -21.92695 | -47.3673 | MH158888 |
| A fasciatus ce46 2 | PRB | -21.92695 | -47.3673 | MH158889 |
| A fasciatus ce46 3 | PRB | -21.92695 | -47.3673 | MH158890 |
| A fasciatus ce46v | PRB | -21.92695 | -47.3673 | MH158891 |
| A fasciatus ce47 2 | PRB | -21.92695 | -47.3673 | MH158892 |
| A fasciatus ce47 3 | PRB | -21.92695 | -47.3673 | MH158893 |
| A fasciatus ce48 1 | PRB | -21.92695 | -47.3673 | MH158894 |
| A fasciatus ce48 2 | PRB | -21.92695 | -47.3673 | MH158895 |
| A fasciatus ce6 1 | PRB | -21.92695 | -47.3673 | MH158896 |
| A fasciatus mom1 | SFR | -20.09887 | -45.77819 | MH158897 |
| A fasciatus mom2 | SFR | -20.09887 | -45.77819 | MH158898 |
| A fasciatus mom3 | SFR | -20.09887 | -45.77819 | MH158899 |
| A fasciatus of1 | PRB | -22.31834 | -46.40782 | MH158900 |
| A fasciatus of2 | PRB | -22.31834 | -46.40782 | MH158901 |
| A fasciatus par1 | PRB | -19.07712 | -46.34538 | MH158902 |
| A fasciatus par2 | PRB | -19.07712 | -46.34538 | MH158903 |
| A fasciatus pnc1 | PRB | -19.07557 | -46.40918 | MH158904 |
| A fasciatus pnc2 | PRB | -19.07557 | -46.40918 | MH158905 |
| A fasciatus pnc3 | PRB | -19.07557 | -46.40918 | MH158906 |
| A fasciatus pnp1 | PRB | -18.91785 | -46.50574 | MH158907 |
| A fasciatus pnp2 | PRB | -18.91785 | -46.50574 | MH158908 |
| A fasciatus pnp3 | PRB | -18.91785 | -46.50574 | MH158909 |
| A fasciatus ps1 | PRB | -23.81258 | -47.70878 | MH158910 |
| A fasciatus rg1 | PRB | -21.92969 | -44.19385 | MH158911 |
| A fasciatus rg2 | PRB | -21.92969 | -44.19385 | MH158912 |
| A fasciatus rg3 | PRB | -21.92969 | -44.19385 | MH158913 |
| A fasciatus sa46 1 | PRB | -23.52329 | -45.86255 | MH158914 |
| A fasciatus sa46 2 | PRB | -23.52329 | -45.86255 | MH158915 |
| A fasciatus sa46 3 | PRB | -23.52329 | -45.86255 | MH158916 |
| A fasciatus sa46 4 | PRB | -23.52329 | -45.86255 | MH158917 |
| A fasciatus sa48 1 | PRB | -23.52329 | -45.86255 | MH158918 |
| A fasciatus sa48 2 | PRB | -23.52329 | -45.86255 | MH158919 |
| A fasciatus sa48 3 | PRB | -23.52329 | -45.86255 | MH158920 |
| A fasciatus sa49 | PRB | -23.52329 | -45.86255 | MH158921 |
| A fasciatus sa50 | PRB | -23.52329 | -45.86255 | MH158922 |
| A fasciatus sfpa | SFR | -20.23446 | -44.26258 | MH158923 |
| A fasciatus sfsc1 | SFR | -17.97626 | -46.84329 | MH158924 |
| A fasciatus sfsc2 | SFR | -17.97626 | -46.84329 | MH158925 |
| A giton 1 | SEC | -23.04052 | -44.93408 | MH158926 |
| A giton 2 | SEC | -23.04052 | -44.93408 | MH158927 |
| A giton 3 | SEC | -23.04052 | -44.93408 | MH158928 |
| A hastatus ang1 | SEC | -22.88861 | -44.27786 | MH158929 |
| A hastatus ang2 | SEC | -22.88861 | -44.27786 | MH158930 |
| A hastatus ang3 | SEC | -22.88861 | -44.27786 | MH158931 |
| A hastatus ang4 | SEC | -22.88861 | -44.27786 | MH158932 |
| A hastatus ang5 | SEC | -22.88861 | -44.27786 | MH158933 |
| A hastatus ang6 | SEC | -22.88861 | -44.27786 | MH158934 |
| A hastatus pf1 | SEC | -22.56106 | -42.68135 | MH158935 |
| A hastatus pf2 | SEC | -22.56106 | -42.68135 | MH158936 |
| A hastatus pf3 | SEC | -22.56106 | -42.68135 | MH158937 |
| A hastatus pf4 | SEC | -22.56106 | -42.68135 | MH158938 |
| A hastatus pf5 | SEC | -22.56106 | -42.68135 | MH158939 |
| A hastatus pr1 | SEC | -22.48463 | -42.66048 | MH158940 |
| A hastatus pr2 | SEC | -22.48463 | -42.66048 | MH158941 |
| A hastatus pr3 | SEC | -22.48463 | -42.66048 | MH158942 |
| A hastatus pr4 | SEC | -22.48463 | -42.66048 | MH158943 |
| A hastatus pr5 | SEC | -22.48463 | -42.66048 | MH158944 |
| A hastatus rm1 | SEC | -22.46395 | -42.65473 | MH158945 |
| A hastatus rm2 | SEC | -22.46395 | -42.65473 | MH158946 |
| A hastatus rm3 | SEC | -22.46395 | -42.65473 | MH158947 |
| A hastatus rm4 | SEC | -22.46395 | -42.65473 | MH158948 |
| A hastatus rm5 | SEC | -22.46395 | -42.65473 | MH158949 |
| A hastatus yp1 | SEC | -22.6364 | -42.71215 | MH158950 |
| A hastatus yp2 | SEC | -22.6364 | -42.71215 | MH158951 |
| A hastatus yp3 | SEC | -22.6364 | -42.71215 | MH158952 |
| A intermedius 1 | SEC | -22.87063 | -44.85053 | MH158953 |
| A intermedius 2 | SEC | -22.87063 | -44.85053 | MH158954 |
| A jacuhiensis 1 | URB | unknown | unknown | MH158955 |
| A jacuhiensis 2 | URB | unknown | unknown | MH158956 |
| A janeiroensis | PRB | unknown | unknown | MH158957 |
| A lacustris ara1 | SFR | -20.12317 | -45.98089 | MH158958 |
| A lacustris ara2 | SFR | -20.12317 | -45.98089 | MH158959 |
| A lacustris ara3 | SFR | -20.12317 | -45.98089 | MH158960 |
| A lacustris boi | SFR | -18.35294 | -45.09293 | MH158961 |
| A lacustris tm1 | SFR | -18.32183 | -45.10911 | MH158962 |
| A lacustris tm2 | SFR | -18.32183 | -45.10911 | MH158963 |
| A lacustris tm3 | SFR | -18.32183 | -45.10911 | MH158964 |
| A mexicanus 1 | MEX | aquarium | aquarium | MH158965 |
| A mexicanus 2 | MEX | aquarium | aquarium | MH158966 |
| A mexicanus 3 | MEX | aquarium | aquarium | MH158967 |
| A mexicanus 4 | MEX | aquarium | aquarium | MH158968 |
| A mexicanus mex | MEX | unknown | unknown | MH158969 |
| A paranae od1 | PRB | -19.23751 | -46.20656 | MH158970 |
| A paranae od2 | PRB | -19.23751 | -46.20656 | MH158971 |
| A paranae od3 | PRB | -19.23751 | -46.20656 | MH158972 |
| A paranae pd1 | PRB | -19.17643 | -46.24495 | MH158973 |
| A paranae pd2 | PRB | -19.17643 | -46.24495 | MH158974 |
| A paranae pd3 | PRB | -19.17643 | -46.24495 | MH158975 |
| A ribeirae pg1 | SEC | -24.5934 | -47.8776 | MH158976 |
| A ribeirae pg2 | SEC | -24.5934 | -47.8776 | MH158977 |
| A ribeirae pg3 | SEC | -24.5934 | -47.8776 | MH158978 |
| A ribeirae pp1 | SEC | -24.3113 | -47.89213 | MH158979 |
| A ribeirae pp2 | SEC | -24.3113 | -47.89213 | MH158980 |
| A ribeirae pp3 | SEC | -24.3113 | -47.89213 | MH158981 |
| A rivularis ab1 | SFR | -19.20991 | -46.10938 | MH158982 |
| A rivularis ab2 | SFR | -19.20991 | -46.10938 | MH158983 |
| A rivularis boi1 | SFR | -18.35294 | -45.09293 | MH158984 |
| A rivularis boi2 | SFR | -18.35294 | -45.09293 | MH158985 |
| A rivularis boi3 | SFR | -18.35294 | -45.09293 | MH158986 |
| A rivularis ct1 | SFR | -18.94279 | -45.93838 | MH158987 |
| A rivularis ct2 | SFR | -18.94279 | -45.93838 | MH158988 |

Hydrographic basins: PRB – Paraná River Basin; SFR – São Francisco River Basin; SEC – Southeast Coastal; URB – Uruguay River Basin; MEX – Mexico.
